# Relevance of food allergy in the assessment of NSAID-involved reactions

**DOI:** 10.1186/2045-7022-5-S3-O22

**Published:** 2015-03-30

**Authors:** Jaime Sánchez-López, Alba García-Moral, Victoria Cardona, Mar Guilarte, Rosa Muñoz-Cano, Mariona Pascal, Maria Rueda, César Picado, Antonio Valero, Joan Bartra

**Affiliations:** 1 Hospital Clínic, Barcelona, Spain; 2 Hospital Vall d’Hebron, Barcelona, Spain

## Background

Nonsteroidal anti-inflammatory drugs (NSAID) are the most frequently involved drugs in hypersensitivity reactions. NSAIDs have also been described as food allergy enhancers. The aim of the study was to evaluate the prevalence of NSAID-enhanced food allergy in a group of patients consulting for an adverse drug reaction to NSAID.

## Method

We prospectively recruited all consecutive patients from June 2012 to May 2014 consulting for an NSAID adverse reaction in which a confirmation diagnosis was reached. The practical approach to the diagnosis of hypersensitivity to NSAIDs was performed according to the protocol from the EAACI Task Force on NSAIDs Hypersensitivity. SPTs were also performed to a standard panel of inhalant and food allergens. Food allergy was assessed by a consistent clinical history and a positive SPT to the foods involved. A subgroup of patients diagnosed as NSAID-enhanced food allergy was challenged with the responsible food in the presence and absence of the NSAID to demonstrate the enhanced effect.

## Results

170 patients were included, mean age 47±15 years, 63% female. 52/170 (31%) were sensitized to any food SPT, being the lipid transfer protein (LTP) the most frequent sensitization (32/52, 61%), followed by shrimp (13/52, 25%). Among them, 36/52 had a previous known food allergy, 2/52 were newly diagnosed and in 14/52 the positive food SPT had no clinical relevance. Within the food allergy group (38 patients), 71% had NSAID-enhanced food allergy, 21% had an NSAID intolerance, 5% had an NSAID selective allergy and 3% NSAID allergy/intolerance was discarded and food allergy had nothing to do within the adverse reaction. The allergens responsible for the NSAID-enhanced food allergy were LTP in 88.9% and gliadin in 11.1%. Globally, NSAID-enhanced food allergy has a prevalence of 16% (27/170) among all the evaluated patients.

## Conclusion

Food allergy should be evaluated routinely in patients with the suspicion of adverse drug reactions to NSAIDs. When food allergy is present, >70% of cases the NSAID enhanced the food reaction. LTP is the main responsible in NSAID-enhanced food allergy in our population.

